# Response to Lea *et al.*’s developmental plasticity: Bridging research in evolution and human health

**DOI:** 10.1093/emph/eox021

**Published:** 2018-02-05

**Authors:** Sylvia Kirchengast

**Affiliations:** Department of Anthropology, University of Vienna, Vienna A-1090, Austria

**Keywords:** developmental plasticity, human life history, growth patterns, maturation

My comments on Lea *et al.*’s review discussing “developmental plasticity: bridging research in evolution human health” will focus on the implications of developmental plasticity on human life history parameters. Today, it is well-known that members of the same sex, age and even species differ dramatically in anatomical, physiological and behavioral traits and that this kind of variation is due to that each individual has the capacity respond to environmental circumstances in more than one way [1]. Consequently a given genotype may generate multiple phenotypes depending on environmental conditions experienced by the organism during critical phases of individual development [2]. Of special importance are early life circumstances which clearly have a profound impact not only on phenotypic parameters and behavior but also on life history and health and disease during later life. Scientific community should receive with satisfaction the review paper of Lea *et al.* since it provides an impressive overview of evolutionary explanations for developing plasticity, and discussed genetic, genomic and molecular mechanism of developmental plasticity extensively. Although so many important topics were treated in the review, the importance of developmental plasticity for human life history traits however was neglected. Therefore I would like to comment about the effects of developmental plasticity on selected traits of human life history. From the viewpoint of human health research, one of the main research questions that enlivened the debate on the long-term consequences of the interplay between the developing organism and the circumstances in which it finds itself is in which way early environmental circumstances enhances or hamper human growth, maturation and reproductive phase, three important parameters of human life history with important implications for public health research [3]. Especially important criteria for health and well-being of children are growth patterns and maturation. Consequently, the analysis of the impact of developmental plasticity on individual differences in growth patterns provide crucial information for the detection of pathologic conditions and may be long-term health effects. Developmental plasticity and life history events have been interpreted from the viewpoint of the developmental constraints model as well as predictive model. As evolutionary biologists we have to be aware that human life history differs not only characteristically in some features from other mammals and even from that of our next living relatives the great apes, life history parameters may vary according to early environmental conditions [4]. The importance of intrauterine environment on fetal growth and also long-term health effects was first formulated in the so-called thrifty phenotype hypothesis [5]. It was assumed that human development may involve induction of particular patterns of development by cues that prepare the developing individual for a distinct type of environment in which it is likely to live in later life. Special problems arise when the environmental prediction provided by the intrauterine environment and the conditions of later life differ. Although this hypothesis has been discussed critically, there is no doubt that adverse environmental circumstances during intrauterine phase may change the trajectory of fetal development and may slow growth. The main consequence is the birth of a small for gestational age newborn. Cohort studies showed that a low birth weight is significantly associated with an increased risk of obesity, diabetes Type 2, hypertension, coronary heart disease and osteoporosis during later life [5, 6]. Furthermore, developmental plasticity and the response to environmental conditions during early development have important fitness consequences because they may lead to long-term modifications in important parameters of human life history. Especially the growth process during infancy and childhood mirrors the interaction between intrauterine environment and environmental conditions during postnatal growth [7]. Human growth is an extremely complex process driven by both genetic inheritance and also by environmental conditions. Small for gestational age newborn may be adapted to an environment characterized by energetic shortages, an improvement of the conditions during infancy and childhood, however, may result in the so-called catch-up growth. This has implications of later reproductive performance. Small for gestational age girls show early adrenarche, early menarche, low ovulation rates and early menopause [8]. Furthermore adrenal androgen secretion is advanced among SGA girls, a hormonal situation which is responsible for secondary sexual characteristics, but also hyperinsulinemia and hyperandrogenemia [8]. Consequently low birth weight but mainly in combination with postnatal catch-up growth resulting in higher body weight and weight gain in infancy and childhood may increase the likelihood of early menarche [9]. Early menarche may by a strategy to enhance the chance of successful reproduction even under adverse environmental conditions. On the other hand, successful female reproduction depends on sufficient body size, first of all sufficient pelvic dimensions and energy deposits such as fat stores. Therefore, an early menarche is only possible when developmental plasticity allows to compensate adverse intrauterine conditions resulting in low birth weight by a catch-up growth during childhood and juvenile phase. Intrauterine conditions and developmental plasticity have also effects on the terminations of reproductive phase among human females. Menopause, the last spontaneous uterine bleeding and postmenopause, i.e. the prolonged postreproductive phase is typical life history characteristics of female *Homo sapiens*. Adverse intrauterine conditions may reduce the number of oocytes and primordial follicle and may lead to earlier menopause. Tom *et al.* [10] reported that low birthweight and higher birthweight standardized by gestational age were associated with earlier age at menopause. These aspects seem to support the developmental constraints model. In general, we can conclude that developmental plasticity has profound impact on human life history parameters, reproductive fitness and consequently health parameters and public health.

**Conflict of interest:** None declared.
